# Pulmonary Arterial Hypertension among HIV-Infected Children: Results of a National Survey and Review of the Literature

**DOI:** 10.3389/fped.2015.00025

**Published:** 2015-04-07

**Authors:** Arnaud Grégoire L’Huillier, Klara Maria Posfay-Barbe, Hiba Pictet, Maurice Beghetti

**Affiliations:** ^1^Pediatric Infectious Diseases Unit, Department of Pediatrics, Geneva University Hospitals, Geneva, Switzerland; ^2^Faculty of Medicine, University of Geneva, Geneva, Switzerland; ^3^Pediatric Cardiology Unit, Department of Pediatrics, Geneva University Hospitals, Geneva, Switzerland

**Keywords:** human immunodeficiency virus, pediatrics, pulmonary artery hypertension, screening, survey

## Abstract

Since the advent of highly active anti-retroviral therapy, HIV-related mortality has decreased dramatically. As a consequence, patients are living longer, and HIV infection is becoming a chronic disease. Patients and caretakers have to deal with chronic complications of infection and treatment, such as cardiovascular diseases, which now represent an important health issue, even in the pediatric population. Prevalence of pulmonary arterial hypertension (PAH) in the adult HIV population is around 0.4–0.6%, which is around 1000- to 2500-fold more prevalent than in the general population. In recent adult PAH registries, HIV has been identified as the fourth cause of PAH, accounting for approximately 6–7% of cases. Therefore, regular screening is recommended in HIV-infected adults by many experts. If HIV-associated PAH is mainly reported in HIV-infected adults, pediatric cases have also been, albeit rarely, described. This scarcity may be due to a very low PAH prevalence, or due to the lack of systematic cardiovascular screening in pediatric patients. As PAH may manifest only years or decades after infection, a systematic screening should perhaps also be recommended to HIV-infected children. In this context, we retrospectively looked for PAH screening in children included in our national Swiss Mother and Child HIV cohort study. A questionnaire was sent to all pediatric infectious disease specialists taking care of HIV-infected children in the cohort. The questions tried to identify symptoms suggestive of cardiovascular risk factors and asked which screening test was performed. In the 71 HIV-infected children for which we obtained an answer, no child was known for PAH. However, only two had been screened for PAH, and the diagnosis was not confirmed. In conclusion, PAH in HIV-infected children is possibly underestimated due to lack of screening. Systematic echocardiographic evaluation should be performed in HIV-infected children.

## Review

### Introduction

WHO estimated in 2010 that 34 millions of humans were infected with HIV, 10% being younger than 15 years old (1). In children, infection mainly happens through transmission between mother and child during the pre-, peri-, or postnatal period (through breastfeeding). The risk of infection differs according to the mode of transmission (2), and also depends on the viremia of the source patient (3). The infected person often presents an asymptomatic period lasting 5–10 years without treatment, depending on viremia and CD4 T-cell count (4).

Since the advent of highly active anti-retroviral therapy (HAART), HIV-related mortality has decreased dramatically both in adults (5) and children (6–8). As a consequence, patients are living longer, and HIV infection is becoming a chronic disease. Patients and caretakers have to deal with chronic complications of the infection and the treatment, such as cardiovascular disease, which now represent an important health issue in this patient population (9).

Because of the lack of randomized control studies, it is difficult to differentiate the etiological role of the virus itself from the impact of HAART in pulmonary arterial hypertension (PAH), as well as other cardiovascular manifestations. PAH is a serious disease of the pulmonary arteries characterized by vascular remodeling due to dysfunctional proliferation of smooth muscle and endothelial cells (10). It leads to an increase in pulmonary vascular resistance and pulmonary arterial pressure (PAP). PAH classification was updated during the World Symposium on Pulmonary Hypertension in 2013 (11). This classification gives more importance to pediatric PAH compared to the previous Danapoint Classification (12).

Despite this, no systematic cardiovascular screening is usually suggested to HIV-infected children. To demonstrate this, we sent a questionnaire to all infectious diseases specialists taking care of HIV-infected children in Switzerland, part of the Swiss Mother and Child HIV (MoCHIV) cohort study to evaluate if they look for PAH symptoms and perform screening procedures. We will first review HIV-related PAH (HIV-PAH) and second describe the results of our questionnaire.

### Epidemiology

Pulmonary arterial hypertension was described in HIV-infected patients for the first time in 1987 (13). Since then, HIV has been identified as one of the associated forms of PAH, accounting for approximately 6–8% of cases (14–17). However, because of the high HIV prevalence worldwide and the poor screening, HIV-PAH may be an important unrecognized cause of PAH. Even if confounding factors, such as drug use and co-infection with hepatitis C virus (HCV) have been reported (18), more than 80% of PAH cases in the HIV population are directly related to HIV and/or its treatment (19, 20).

Pulmonary arterial hypertension is defined as a resting PAP >25 mmHg, associated with a pulmonary arterial occlusion pressure (PAOP) ≤15 mmHg (21). HIV-PAH diagnosis can only be confirmed once all other possible etiologies have been excluded.

Diagnosis relies on clinical symptoms, such as fatigue and dyspnea, as well as clinical findings, such as an increase in the pulmonary component of the second heart sound, a tricuspid regurgitation murmur or a right fourth heart sound, and signs of right heart failure. Chest X-rays may show cardiomegaly and pulmonary arteries enlargement, and the electrocardiogram shows right ventricular hypertrophy with right axis deviation. When PAH is suspected, echocardiography is the most useful diagnostic tool (22). Confirmation with right heart catheterization (RHC) is the gold standard. Some authors recently proposed a diagnosis algorithm (23, 24).

Prevalence of HIV-PAH is around 0.2–0.6% (20, 23, 25–30), which is 1000- to 2500-fold more prevalent than in the general population (20, 23). However, as PAH may present with very scarce symptoms and no systematic screening is usually offered in the HIV-infected population, these results are probably underestimated. Most previously mentioned studies included only symptomatic patients. When all HIV-infected patients of a cohort are included and/or when using only echocardiography without RHC confirmation, HIV-PAH prevalence reaches 2.5–10% (18, 29, 31–33). Compared to patients with HIV-PAH diagnosed using RHC, mean PAP values were 20 mmHg higher when diagnosis was established only with echocardiography, suggesting that echocardiography overestimates PAP (22). Hsue et al. described pathological PAP by echocardiography in 35% of their HIV-infected cohort (14). In a recent study, HIV-PAH prevalence was 57%, but only patients with tricuspid regurgitation were included and methods to calculate PAP were different (34).

### Physiopathology and histological features

HIV-related PAH physiopathology is not completely understood, is probably multifactorial, and includes genetic factors. It is hypothesized that HIV acts as a trigger, maintaining chronic inflammation and immune activation. Histological features, which closely resemble those seen in idiopathic PAH, show in most cases a pulmonary arteriopathy with so-called “plexiform lesions” associated with concentric laminar intimal fibrosis, medial hyperplasia, and white cells. They suggest chronic inflammation (13, 19, 20, 28, 35–38). Occasionally, plexiform lesions are lacking, possibly because it is an earlier stage of the disease (39). More rarely, thrombotic arteriopathy of the small vessels has been described (40). As simian immunodeficiency virus (SIV) infection of macaques shares many characteristics with HIV infection, these animals have been used as non-human primate model of HIV: plexiform lesions were only recovered among macaques infected with SHIV, a viral construct containing the HIV Nef protein in an SIV backbone, but not among SIV alone-infected macaques (41). In contrast, in another animal study, all SIV and SHIV macaques had elevated PAP associated with histological changes consisting mostly of intimal and medial hyperplasia. PAP values were higher in SIV and SHIV macaques than in healthy macaques, and PAP increased as early as 3 months after SIV infection (42).

HIV was never detected – to our knowledge – in the pulmonary endothelial cell using different techniques, such as immunohistochemistry, HIV-DNA hybridization, electron microscopy, or PCR. The absence of virus in endothelial cells, but its presence in alveolar macrophages, suggests an indirect action through mediator release rather than a direct endothelial infection (39, 43–45). Absence of virus in endothelial cells has also been described in SIV (46).

Endothelial dysfunction in PAH is characterized by a reduced production of vasodilatators prostacyclin and nitric oxide (NO) and an increased production of endothelin-1 (ET-1) (47), a potent vasoconstrictor: its importance in idiopathic PAH pathology has been widely studied (48). Pellicelli et al. demonstrated increased levels of ET-1, IL-6, and TNF-alpha among HIV-PAH patients compared to HIV-infected patients without PAH. They also showed that plasmatic concentrations of ET-1 and IL-6 were correlated to severity of HIV-PAH (49).

The role of viral proteins has also been studied. Despite the absence of virus in endothelial cells, HIV envelope protein Gp120 has been shown to be toxic *in vitro* by inducing pulmonary endothelial cells apoptosis and time- and dose-dependent increase in ET-1 (50, 51). Moreover, Gp120 activates T lymphocytes (52) and increases TNF-alpha production (51), which sustains inflammation and may play a subsequent role in HIV-PAH development. Another recent study has shown that Gp120 stimulates arterial smooth muscle cells to a express tissue factor, initiating the coagulation cascade (53). Other HIV viral proteins, such as Nef and Tat, lead to chronic inflammation and endothelial dysfunction and may play a role in HIV-PAH pathogenesis. Nef has been shown to increase monocyte migration, enhancing local inflammation (54). Moreover, Nef was detected in lungs endothelial cells from HIV-PAH patients, but not among healthy or idiopathic PAH patients, suggesting that while the virus does not enter endothelium, it is possible that secreted viral proteins do (41). A group recently showed that the risk of HIV-PAH was related to the number of Nef mutations and that some polymorphisms mapped to Nef functional domains were overrepresented among HIV-PAH patients (55, 56).

Tat is another viral protein secreted by HIV-infected cells, which stimulates endothelial cells, enhancing vascular permeability via IL-6 (57). It has been suggested that Tat induces chronic inflammation and endothelial dysfunction (58). Once the endothelium is damaged, exposure of vascular smooth muscle cells to viral proteins such as Tat down regulates antiangiogenic factor BMPR: this promotes vascular smooth muscle cells proliferation and hypertrophy (59).

Similarly, HIV-1 proteins expression increased pulmonary vascular resistance among rats exposed to chronic hypoxia compared to wild-type rats (60).

Levels of asymmetric dimethylarginine (ADMA), an endothelial NO synthase inhibitor, are increased during HIV infection because of sustained inflammation. This may contribute to endothelial dysfunction, as ADMA levels are correlated with PAP in HIV-infected patients (61). Platelet-derived growth factor (PDGF) can induce proliferation and migration of smooth cells: Humbert et al. found that PDGF expression was increased in perivascular areas of the lungs of HIV-PAH patients (43). Similarly, increased vascular endothelial growth factor (VEGF) expression in HIV-infected patients may alter vascular permeability and induce endothelial cells (62). As only a small proportion of patients develop HIV-PAH, a genetic predisposition is assumed, such as the reported association between HIV-PAH and HLA DR6 and DR52 alleles (63). Despite being identified in 70% of familial PAH and 20% of idiopathic PAH, no BMPR-2 mutation has been identified among HIV-PAH patients (25).

Other factors may contribute to HIV-PAH pathophysiology. HCV infection or drug use are independent etiologies of PAH; however, as they are more frequent among HIV-infected patients, they may act as confounding factors (18, 20). Cool et al. showed the presence of human herpes virus-8 (HHV-8) in lung lesions of patients with idiopathic PAH (64). This was not confirmed in other studies on idiopathic PAH patients (65) and in HIV-PAH patients, despite their immunosuppression (14, 66). Immune restoration in mice infected by *Pneumocystis jirovecii* has been shown to increase the risk of persistent PAH. However, the persistence of PAH after treatment suggests that chronic inflammation is the most important factor rather than acute infection itself (67).

### Clinical presentation

Clinically, symptoms of HIV-PAH are similar to idiopathic PAH. Early HIV-PAH may be misdiagnosed because of non-specific symptoms, such as exercise intolerance, progressive dyspnea (85%), edema (30%), and non-productive cough (19%) (19). With the progression of the disease, signs of right ventricular dysfunction appear. Compared to idiopathic PAH, patients reported in the literature are usually younger (mean age 33 years) (19) and less symptomatic (35, 68). Males are proportionally more affected (male: female ratio 1.5:1 compared to 1:1.7 in idiopathic PAH), reflecting the prevalence of the HIV population (19). Moreover, time between symptoms and diagnosis is generally shorter than in idiopathic PAH: possibly a consequence of closer follow-up (25).

HIV-related PAH carries a poor prognosis: mortality is mainly due to PAH rather than to HIV complications: PAH is therefore a predictor of mortality by itself (20, 25, 26, 68). Initial studies reported a median survival between 0.5 and 2.7 years (19, 27), and only 21–46% median survival at 3 years (19, 25–27). Earlier studies reported a faster progression (35) and a shorter survival with HIV-PAH compared to idiopathic PAH (20, 35). This could be explained by a more prevalent and severe ventricular systolic dysfunction among HIV-PAH patients compared to patients with PAH of other etiologies (69). More recent studies have shown a slightly improved median survival with 66–86% at 3 years (17, 70, 71). As many deaths in these recent studies were unrelated to PAH, one may suppose a decrease in HIV-PAH-related death (71). Survival significantly decreases with an increase of NYHA stages; therefore, a clinical marker of HIV-PAH progression (25, 68), would be crucial to identify patients in the early, scarcely symptomatic phase of the disease.

The relationship between the severity of HIV and PAH is subject to debate. HIV-PAH occurs at all stages of HIV infection. Most authors could not show an association between HIV-PAH and low CD4 T cell count (19, 20, 23, 27, 28, 34, 68), even if the proportion of patients with CD4 count <200/mm^3^ was higher among HIV-PAH patients in one study (23). On the other hand, other groups described an increased survival rate with increasing CD4 counts (17, 25, 26).

Regarding viremia, several studies could not find an association with HIV-PAH (23, 34, 68) but others described an increased survival with lower viremia (17, 71). Finally, in one study, viremia was higher among HIV-PAH patients than among HIV patients without PAH (18). To date, there are no sufficient data to confirm a protective role of HIV control through HAART on HIV-PAH’s incidence and progression.

### Treatment of HIV-PAH

Because survival is related to NYHA stage, early diagnosis and treatment are paramount (25, 68). There are no current validated guidelines and HIV-PAH treatment is still controversial because of the lack of randomized studies.

#### Non-specific treatment

Oxygen reduces hypoxia-induced pulmonary vasoconstriction, whereas diuretics reduce right ventricular preload, and digoxin increases cardiac output. Oral anticoagulation reduces the possible thrombotic component of PAH (72), but its use for HIV-PAH is often limited due to concomitant thrombocytopenia and hepatopathy. Calcium channel blockers (CCB) use has been disappointing in adult HIV-PAH because almost all patients are not responders to acute vasoreactivity testing, it lacks a sustained vasodilator effect and has important side-effects (26), possibly related to interactions with HAART (73). Given that a positive acute vasoreactivity response to CCB is more frequent in children than adults and that there is no pediatric data concerning CCB use in HIV-PAH, these medications should be considered in selected cases, but vasoreactivity is not common in HIV-PAH.

#### Specific treatment

Prostacyclin analogs can be administered by IV infusion, inhalation, or subcutaneously.

Epoprostenol, the intravenous form of a prostacyclin analog, has been shown to induce clinical (25, 74) and hemodynamic improvement (25, 35, 74), especially when combined to HAART (25). Epoprostenol has been shown to increase survival (25). Its administration mode requires the use of a central venous catheter, which is linked with risks of infection and of misuse in drug abusers. Inhaled iloprost also showed hemodynamic (27, 75, 76) and clinical (75, 76) improvement, albeit not statistically significant. Subcutaneous treprostinil increases hemodynamic and clinical parameters (77) in HIV-PAH patients.

Sildenafil, a phosphodiesterase inhibitor type 5, is the most frequently used medication in idiopathic PAH. In HIV-PAH, its use is limited because of its interaction with protease inhibitors (PIs), through the 3A4 P450 cytochrome pathway (78–80). However, in case reports of patients with HIV-PAH, it showed clinical and hemodynamic improvement (81–83).

Bosentan, an oral dual endothelin receptor antagonist, acts by inhibiting the potent vasoconstricting properties of endothelin. Bosentan has been shown to induce a clinical and hemodynamic improvement among HIV-infected patients (26, 71, 84, 85). Some patients even normalized their hemodynamic values during treatment (71). In 2012, Tcherakian et al. reported two cases of sustained and completely reversible HIV-PAH with bosentan treatment, persisting after drug discontinuation (86). Bosentan is a moderate Cytochrome P450 3A4 and 2C9 inducer, therefore simultaneous administration of anti-retroviral drugs should be performed with caution (87).

A treatment algorithm was recently proposed for HIV-PAH (88).

### Role of HAART in HIV-PAH

The role of HAART in PAH is unclear. As chronic inflammation probably contributes to HIV-PAH pathophysiology, one may suspect a beneficial effect of HAART on HIV-PAH by reducing inflammation. In rats, PIs reverse or decrease PAH progression by reducing remodeling and smooth muscle cell proliferation (89). In humans, some authors showed no change in the frequency or severity of PAH since HAART was introduced, or in patients treated with HAART (17, 18, 23, 26), whereas others described a decrease of PAH incidence (27, 90) associated with a decline in HIV-PAH-related mortality (25, 27, 71). Among patients with HIV-PAH, clinical improvement was observed, especially when associated to PAH medications (17, 85). In a similar fashion, hemodynamics improved with HAART whether or not associated to PAH medication (26, 27, 85, 91). It is possible that PIs may be the most beneficial (27).

On the other hand, other studies have shown a deleterious effect of HAART on HIV-PAH, possibly secondary to PI-induced endothelial dysfunction (92). *In vitro* studies have demonstrated that anti-retrovirals, especially PIs, increased ET-1 production, followed by endothelial and vascular smooth muscle cell proliferation (93). Among rats, administration of AZT and PIs was associated to endothelial dysfunction and an increase in ET-1 production (94). In humans, some authors describe stable HIV-PAH after HAART interruption (95), while others describe an increased incidence with HAART compared to ART (96, 97) or an accelerated HIV-PAH evolution (44). Reinsch et al. reported an increased echocardiographically proven HIV-PAH prevalence among patients treated with HAART compared to naïve patients (31). Another human study associated ritonavir-boosted PIs and abacavir with HIV-PAH (34).

In general, HIV-PAH prevalence has not changed significantly since HAART was introduced. This may suggest that antiviral drugs have a little effect on sites where the virus affects vascular cells. However, a protective effect of HAART on survival and incidence may explain a stable prevalence, as patients are now living longer. In clinical practice, since the Opravil et al. study showing an improvement of the pressure gradient when the patients were treated, experts recommend to introduce HAART at the time of HIV-PAH diagnosis, regardless of CD4 T-cell counts (26).

### Pediatric data

While HIV-PAH mainly concerns HIV-infected adults, pediatric cases have also been described (63, 83, 98–104), the youngest being between 1 and 2 years old (83, 102). Apart from HIV infection, few of these children also have other PAH predisposing factors, such as hemophilia (99, 101). The youngest patient is an HIV-infected very-low birth-weight infant, which presented with neonatal PAH: his condition resolved with HAART treatment but confounding factors, such as persistent ductus arteriosus, prolonged mechanical ventilation, extreme prematurity, systemic infection, and persistent pulmonary hypertension of the newborn, may have contributed to the disease (105). The causality is therefore uncertain. In a cohort of pediatric PAH patients in the Netherlands, 1% of cases were infected with HIV (98). In vertically HIV-infected children in Thailand, more than 40% presented echocardiographic criteria for HIV-PAH, but in more than half of the cases, this could be explained by lymphoid interstitial pneumonia (106). More recently, Ferrand et al. diagnosed HIV-PAH by echocardiography in 7% of vertically infected adolescents (aged 10–19 years) from Zimbabwe (107). Cunha et al. described an echocardiographic prevalence of HIV-PAH in 4.4% of a Brazilian children cohort younger than 13 years (108). Two studies in Thailand and Zimbabwe including only symptomatic HIV-infected toddlers have described an HIV-PAH prevalence of 48–75% (109, 110). The prevalence of echocardiographic HIV-PAH among children is similar or even higher than in adult reports using only echocardiography as a diagnostic tool. This high prevalence among HIV-infected children in Asia, Africa, and South America may be explained by vertical transmission. A delayed HIV diagnosis such as described in Ferrand et al. (median age at diagnosis 12 years) may also have contributed to the high HIV-PAH prevalence (107). Finally, confounding factors, such as high prevalence of parasitic diseases, chronic hepatitis B and C infection, and high seroprevalence of HHV-8 could have played a role.

In a recent registry study of more than 360 children in 19 countries – including Switzerland – with confirmed PAH, there was no HIV-infected child (111). Similarly, there was no HIV-infected patient in another registry study including more than 200 children with PAH (112). This may be due to the fact that no systematic screening is performed in the HIV-infected population but also to a selection bias of both registries and their participating centers as almost all patients were symptomatic.

## What is Done in Switzerland?

To demonstrate a correlation between HIV and PAH in HIV-infected children, we retrospectively analyzed cardiovascular and, in particular, PAH screening of children in the Swiss MoCHIV cohort study.

The MoCHIV Cohort Study is a merger of the former Neonatal HIV Study and the Swiss HIV and Pregnancy Study. Initially, the main goal was to collect a maximum of data in children born to HIV-infected mothers and HIV-infected children to gain epidemiological information and to study vertical transmission and natural history of disease. In 1998, a merged project was built named The Swiss MoCHIV Study. In 2004, a permanent link was established between the Swiss HIV Cohort Study (SHCS) and MoCHIV. Thus, longitudinal data in women included in both cohorts became available for research, making MoCHIV a very unique mother–child cohort. All mothers and children are given a cohort number, which is then used to collect data in both cohorts anonymously. Women and children are followed according to good clinical practices, as defined by the respective guidelines for adults, pregnant women, and children. These have been regularly adjusted according to growing knowledge.

**Figure 1:**
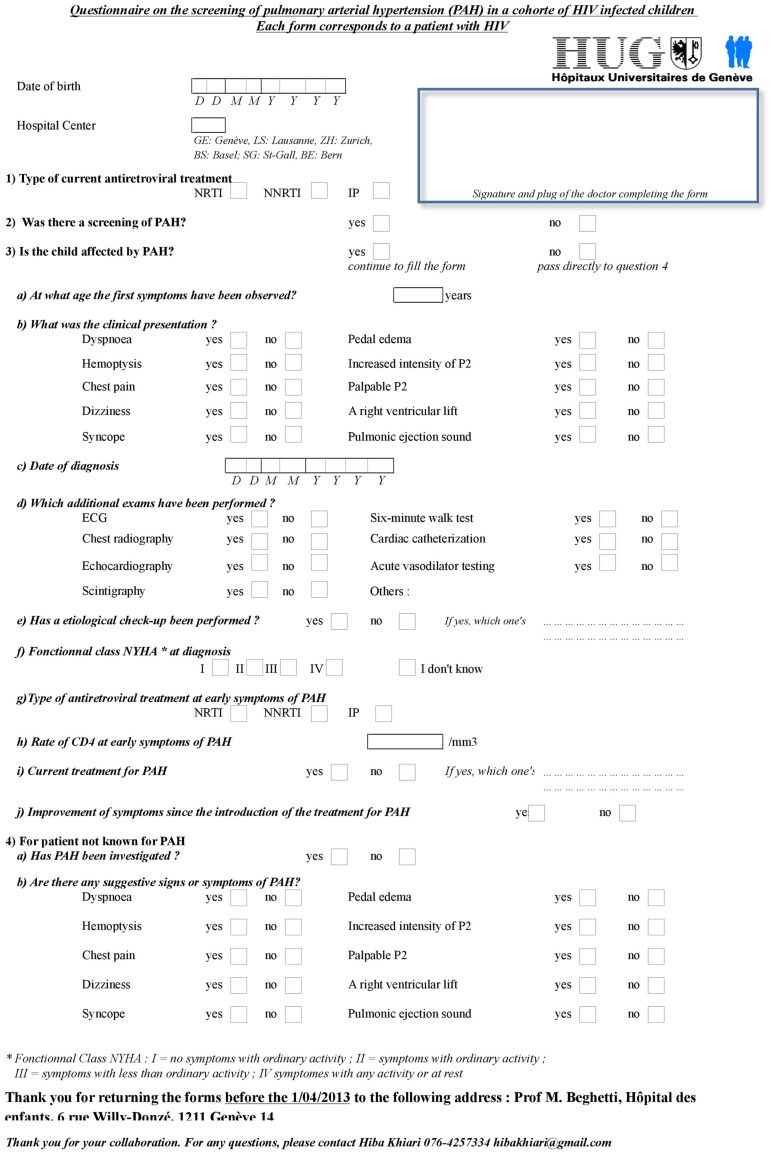
**Questionnaire sent to Swiss pediatric infectious diseases specialists following HIV-infected children**.

It is estimated that approximately 75% of HIV-infected children living in Switzerland are part of MoCHIV. All Swiss pediatric infectious disease experts following HIV-infected children in the cohort received a person-specific questionnaire. The questions concerned symptoms suggestive of PAH and which cardiovascular screening was performed. In addition, questions concerning HIV status (CD4 T-cell count, viremia, HAART treatment or not) were recorded (Figure 1).

Among the 83 children currently included in the MoCHIV cohort study, the questionnaire was retrieved for 71 (86%) children. As suspected, no child of the MoCHIV cohort was known for PAH. Only two children (3%) had been screened for PAH, one because of thoracic pain and one because of dizziness, but diagnosis was not confirmed. Echocardiographic studies have reported higher prevalence of PAH (106–110). It is therefore possible that HIV-PAH is under-diagnosed in Switzerland as well because systematic screening is not performed. Only symptomatic cases would be (or will be) detected. Alternatively, it is also possible that other cofactors are involved and explain the higher prevalence in other countries.

## Conclusion

Increased PAH risk in HIV-infected children, due to HIV infection and/or anti-retroviral treatment, creates new challenges and has serious implications for the quality of life and the survival of this population. However, despite a clearly increased risk, PAH detection is neglected in HIV-infected children. The true impact of PAH can only be appreciated after years or decades because of its silent and slow progression. Survival among HIV-infected children will probably continue to increase with more potent treatment options. As demonstrated in several studies, the prevalence of echocardiographic signs of increased PAP despite being below PAH definition level is more frequent than expected, even in children. Therefore, a systematic PAH screening should be proposed in this at-risk population (113–117). An echocardiography should be performed in all HIV-infected children at least once. However, a regular echocardiographic follow-up would be even more helpful, despite its cost, because there is a better outcome when diagnosis is early. RHC should be offered to all patients with abnormal echocardiographic findings. This has been successfully done in adult patients with sickle cell disease or scleroderma, another pathology associated with PAH (118, 119). Moreover, because only a proportion of HIV-infected patients develop HIV-PAH, finding biomarkers predicting HIV-PAH would be essential for the future.

## Conflict of Interest Statement

The Review Editor Dunbar Ivy declares that, despite having collaborated on publications in the last 2 years with author Maurice Beghetti, the review process was handled objectively. The authors declare that the research was conducted in the absence of any commercial or financial relationships that could be construed as a potential conflict of interest.
